# Serum microRNA profiles as prognostic biomarkers for HBV-positive hepatocellular carcinoma

**DOI:** 10.18632/oncotarget.10082

**Published:** 2016-06-15

**Authors:** Hao-Tu Zhu, Abdulbaqi M. E. Hasan, Rong-Bin Liu, Zi-Chen Zhang, Xiao Zhang, Jing Wang, Hai-Yun Wang, Fang Wang, Jian-Yong Shao

**Affiliations:** ^1^ State Key Laboratory of Oncology in South China, Collaborative Innovation Center for Cancer Medicine, and Department of Molecular Diagnostics, Sun Yat-sen University Cancer Center, Guangzhou, 510060, China

**Keywords:** serum microRNA, biomarkers, hepatocellular carcinoma, prognosis, deep sequencing

## Abstract

To establish serum microRNA profiles as prognostic biomarkers in hepatocellular carcinoma patients (HCCs), we used deep sequencing to screen serum microRNAs in a discovery set. Twelve up-regulated serum miRNAs were selected for qPCR analysis in a training set. MiR-192-5p and miR-29a-3p were identified and associated with HCC prognosis. HCCs with high concentrations of miR-192-5p and miR-29a-3p had poorer overall survival (OS) and progression-free survival (PFS) than those with low concentrations. We calculated a prognostic index (PI) score and classified patients into low-, medium- and high-risk groups. OS and PFS among the 3 groups from the training set were significantly different (all *P* < 0.05). PI (PI_OS_, PI_PFS_) score was the only independent prognostic predictor for OS and PFS of HCCs in the training set. These results were further confirmed in a validation set. In conclusion, differentially expressed serum miRNAs can be helpful for predicting survival in HCCs.

## INTRODUCTION

Hepatocellular carcinoma (HCC) is the most common and highly malignant hepatoma, the fifth most common cancer, and the third most common cause of cancer-related death in the world [1, 2]. Such a high fatality rate shows the unsatisfactory outcome of HCC and the lack of effective therapeutic strategies for this disease [3], mainly due to frequent postsurgical recurrence and metastasis [4, 5]. Discovery of specific biomarkers for accurate classification and stratification of HCC would be important in improving the prognostic assessment of HCC patients.

The discovery of microRNAs (miRNAs) has opened new avenues for cancer diagnosis, prognosis and prediction of treatment response [6]. miRNA signatures in HCC tissue have been shown to be associated with patient survival [7–9]. miRNAs also circulate in the blood in a cell-free form, stabilised by incorporation into microvesicles or RNA-binding proteins such as Ago2 [10–14]. miRNAs obtained from serum have been reported to provide prognostic information in patients with non-small-cell lung cancer and pancreatic cancer, indicating that serum miRNAs can indeed serve as prognostic markers in cancer patients [15–17].

In fact, differential expression of several miRNAs in the serum, including miR-16, miR-122, miR-21, miR-223, miR-25, miR-375 and let-7f in patients with HCC, patients with hepatitis B, and healthy individuals has been reported recently [18, 19]. However, most of those studies are confined to diagnostic miRNA signatures, and little research has been done to evaluate the prognostic value of circulating miRNAs in HCC. Thus, investigation of aberrant circulating miRNAs as prognostic biomarkers for HCC is an attractive and promising field.

Here, we describe the differential expression of serum miRNAs in 50 hepatitis B virus (HBV)-positive HCCs and 50 healthy controls (HCs) using deep sequencing technology in a discovery set. We used these results to establish a risk model in a training set of 74 HBV-positive HCCs and further validated the risk model in a validation set of 100 HBV-positive HCCs.

## RESULTS

### High-throughput sequencing of small RNAs from HCCs and HCs

To derive optimal serum miRNA profiles, we carried out high-throughput next-generation sequencing of 50 HCC samples and 50 HC samples in the discovery stage. All annotated small RNAs are explained in Supplementary Figure S1. A total of 12 993 179 and 13 117 991 reads were sequenced from 2 small RNA libraries constructed from HC and HCC samples, respectively. From HCs, 11 324 354 clean reads were obtained, which accounted for 87.2% of reads, whereas 11 667 958 clean reads were obtained for HCC samples. We also sought to obtain the sequences of small RNAs shared between the 2 groups (see Supplementary Figure S2A and Supplementary S2B).

### Differential expression of serum miRNAs screened by sequencing in the discovery set

**Figure 1:**
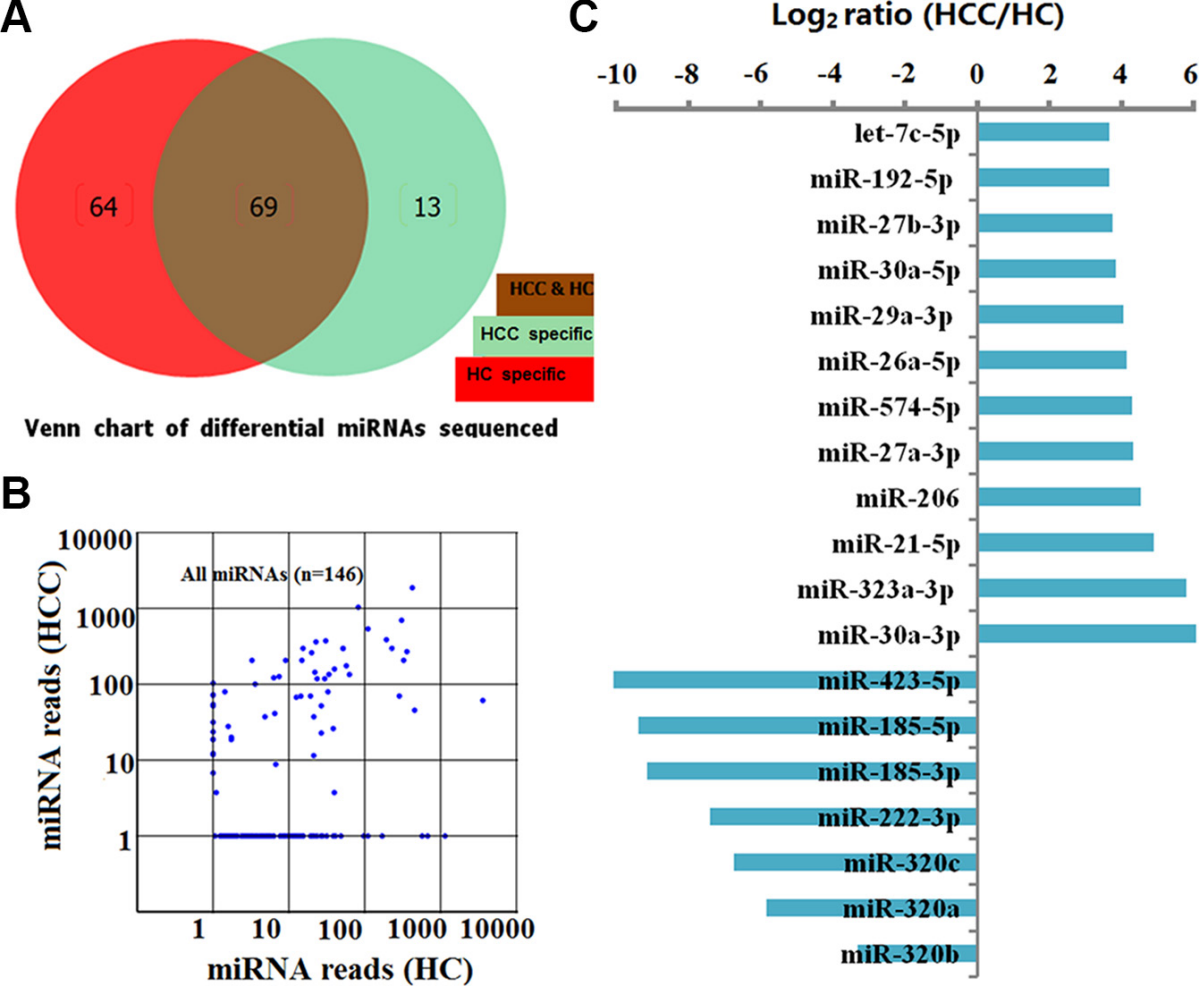
Characterization of 146 known human miRNAs screened by sequencing between HCs and HCCs in the discovery set (**A**) Number and overlap of known human miRNAs between HCs (*n* = 50) and HCCs (*n* = 50). (**B**) Scatter plot of miRNA reads for 146 miRNAs sequenced from 50 HCs and 50 HCCs. Each dot represents 1 miRNA. (**C**) 12 up-regulated and 7 down-regulated miRNAs with significantly different expression levels between HCs and HCCs (miRNA read counts > 100, sequencing-read count ratio > 8 and *P* values < 0.05).

A total of 146 known human miRNAs were found by use of the Illumina/HiSeq2000 platform at the discovery stage (see Supplementary Table S1). Following BLASTN searches and further sequence analysis, 13 miRNAs were found that were unique to HCCs and 64 miRNAs for HCs. In the serum of HCCs and HCs, 69 miRNAs coexisted (Figure 1A and 1B) that had previously been deposited in the miRBase database. On the basis of our criteria (miRNA read counts > 100, sequencing-read count ratio > 8 and *P* values < 0.05), 12 up-regulated and 7 down-regulated miRNAs with significantly different expression levels between HCCs and HCs were identified (Figure 1C). Because we were interested in potential markers that were practical and convenient for predicting HCC prognosis in clinical applications, ultimately we selected 12 miRNAs up-regulated in HCC for further consideration in the training set.

### Correlation of differentially altered serum miRNAs and HCCs prognosis in the training set

**Figure 2:**
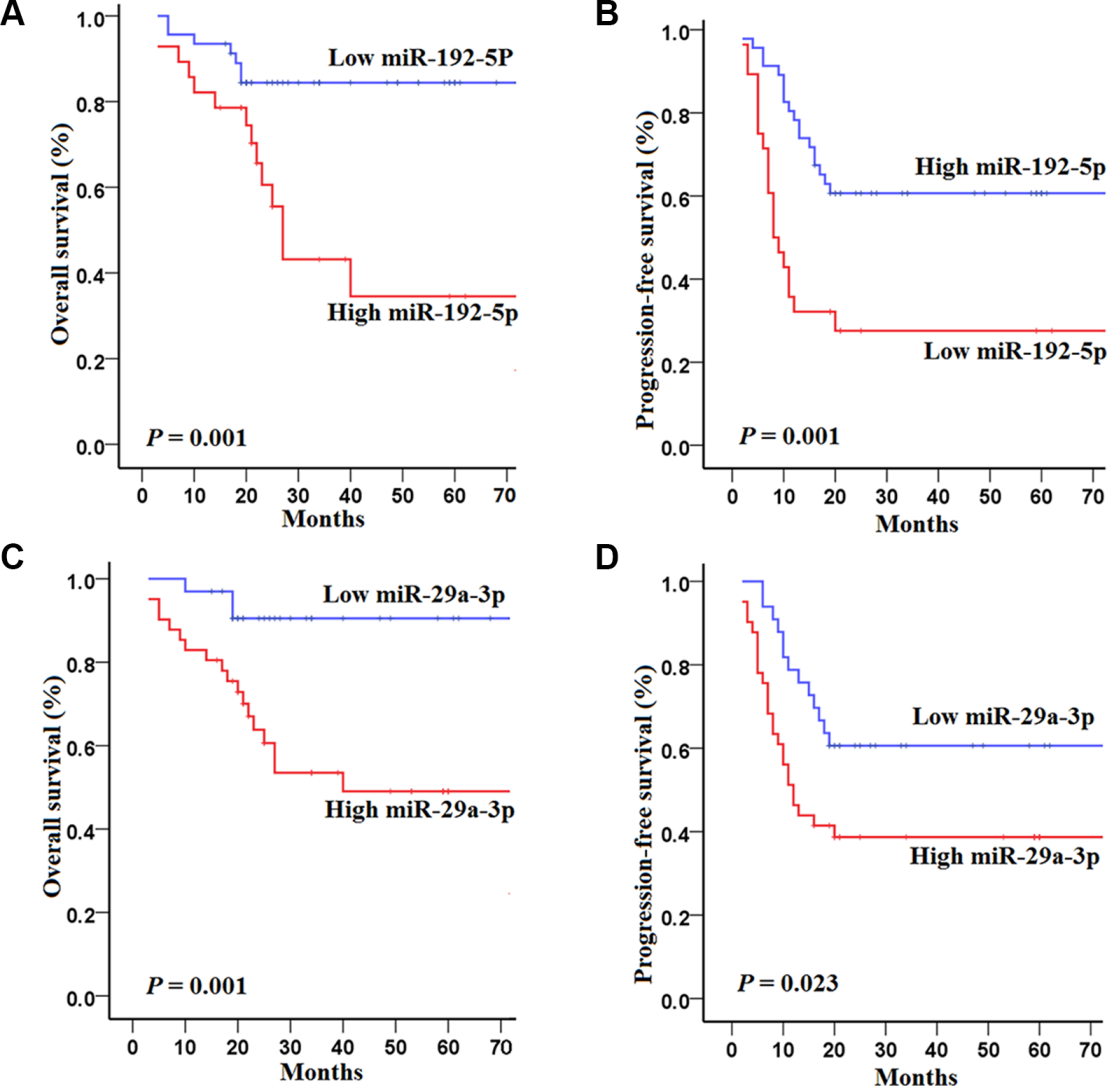
Kaplan–Meier curves of overall survival (OS) and progression-free survival (PFS) for 74 HCC patients in the training set with high and low concentrations of serum miRNAs (**A**–**B**) OS and PFS for 74 HCCs in the training set according to the concentrations of miR-192-5p; (**C**–**D**) OS and PFS for 74 HCCs in the training set according to the concentrations of miR-29a-3p. The survival rates were compared using log-rank test.

To validate the sequencing data, we performed qPCR for each of these 12 up-regulated miRNAs screened by sequencing, and found that only 8 candidate miRNAs (let-7c-5p, miR-192-5p, miR-21-5p, miR-27a-3p, miR-27b-3p, miR-29a-3p, miR-30a-5p and miR-574-5p) were differentially altered between 74 HCCs and 60 HCs in the training set (see in the Supplementary Figure S3). Kaplan–Meier analysis confirmed that miR-192-5p and miR-29a-3p were were associated with HCCs prognosis and progression, while the remaining miRNAs were not found to be significantly predictive for HCCs outcome (Figure 2).

Specifically, HCCs in the training set with high concentrations of miR-192-5p had poorer OS (log-rank test, *P* = 0.001) and PFS (*P* = 0.001) than those with low concentrations of miR-192-5p (Figure 2A and 2B). Similarly, HCCs in the training set with high concentrations of miR-29a-3p had poorer OS (*P* = 0.001) and PFS (*P* = 0.023) than those with low concentrations of miR-29a-3p (Figure 2C and 2D).

A univariate Cox regression model indicated that 6 variables including AFP, Barcelona Clinic Liver Cancer stage (BCLC stage), tumor rize, vascular invasion, miR-192-5p and miR-29a-3p were correlated with the survival of HCCs (see Supplementary Table S2). Subsequently, multivariate analyses revealed that BCLC stage and miR-29a-3p were independently correlated with the OS of HCCs, while AFP, BCLC stage and miR-192-5p were independently correlated with the PFS of HCCs. Taken together, these findings suggest that AFP, BCLC stage, miR-29a-3p and miR-192-5p are independent prognostic factors for survival in the training set (Table 1).

**Table 1:** Multivariate Cox regression analyses of OS and PFS according to clinical variables and the levels of 2 serum miRNAs in the training set

		OS	
Variable	HR^1^	95% CI	*P*
BCLC stage^2^			
(0–A)^3^	1	reference	
(B–C)^4^	5.9	(1.8−18.8)	0.003
miR-29a-3p			
≤ 1.37	1	reference	
> 1.37	4.0	(1.2−13.9)	0.027

		PFS	
Variable	HR	95% CI	*P*
AFP^5^			
≤ 35 ng/ml	1	reference	
> 35 ng/ml	2.1	(1.1−4.2)	0.031
BCLC stage			
(0−A)	1	reference	
(B−C)	5.7	(2.8−11.7)	0.000
miR-192-5p			
≤ 2.24	1	reference	
> 2.24	2.2	(1.1−4.2)	0.023

HR, hazard ratio; BCLC stage, Barcelona Clinic Liver Cancer stage; (0−A), Early stage; (B−C), Late stage; AFP, alpha fetoprotein.

### Dynamic changes of serum miRNAs among HCCs, cirrhosis, and HCs in the training set

**Figure 3:**
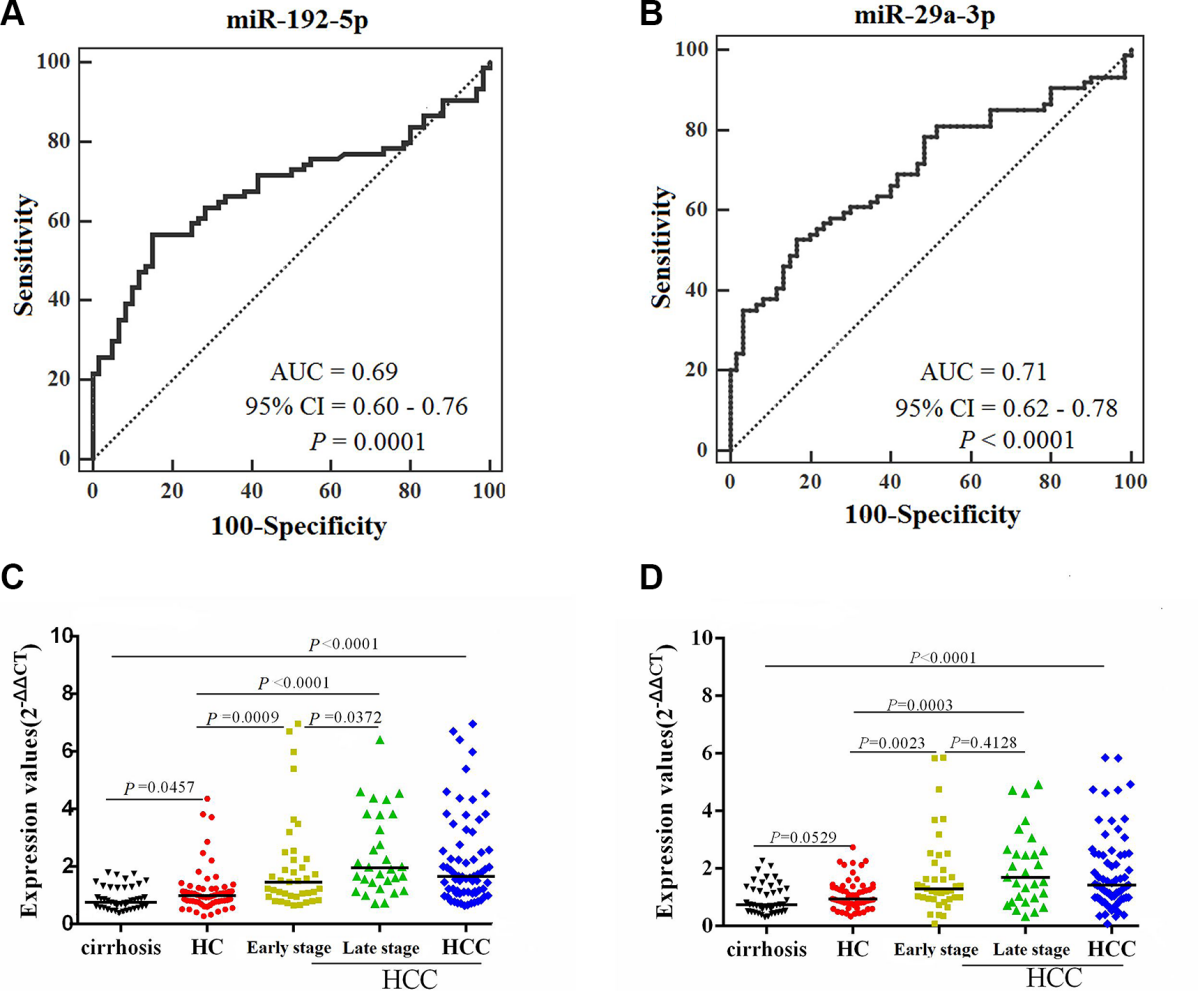
Comparison of the sensitivity and specificity for HCC diagnosis by 2 miRNAs, with scatter plots representing dynamic expression changes of serum miRNAs between controls and HCCs AUCs of miR-192-5p (**A**) and miR-29a-3p (**B**) were analyzed with ROC curves indicating the potential value of the 2 miRNAs to identify HCC. Mann-Whitney unpaired tests using independent samples were used to compare serum miRNA concentrations of miR-192-5p (**C**) and miR-29a-3p (**D**) among the groups of cirrhosis patients (*n* = 43), HC (*n* = 60), early- (*n* = 42) and late-stage HCC (*n* = 32), respectively.

Four of the 8 miRNAs had significantly different expression levels between the HCCs and control groups (healthy and cirrhosis) in the training set, as shown in Table 2. These were let-7c, miR-27b-3p, miR-29a-3p, and miR-192-5p. With respect to the diagnostic value of the 2 miRNAs, a ROC curve was used to distinguish HCCs from HCs in the training set. The AUCs for HCC diagnosis determined from miR-192-5p and miR-29a-3p were 0.69 (95%CI 0.60–0.76, *P* = 0.0001) and 0.71 (95%CI 0.62–0.78, *P* < 0.0001), respectively (Figure 3A−3B).

**Table 2:** Four of the 8 candidate miRNAs had significantly different expression levels between the HCC and control groups^1^ in the training set

NO. miR name		HCC vs Control^1^	HCC vs healthy	fold change	HCC vs cirrhosis	fold change
		*P* value	*P* value		*P* value	
1	**let-7c-5p**	**0.0461**	0.035	1.491	0.119	1.312
2	miR-21-5p	0.0683	0.109	1.212	0.162	1.353
3	**miR-27b-3p**	**0.0001**	0.0002	1.910	0.0001	2.328
4	miR-27a-3p	0.2072	0.045	1.386	0.103	1.117
5	**miR-29a-3p**	**0.0003**	0.031	1.81	0.021	1.901
6	miR-30a-5p	0.2181	0.155	1.112	0.179	1.079
7	**miR-192-5p**	**0.0001**	0.0003	1.711	0.0002	1.973
8	miR-574-5p	0.2114	0.391	1.012	0.172	1.231

Control group includes healthy participants, and patients with cirrhosis.

The median levels of the 2 serum miRNAs were significantly higher in HCCs than in control groups (healthy controls and cirrhosis patients) (Figure 3C−3D). The median levels of the 2 serum miRNAs were significantly higher in both early and late stage HCCs than in controls (Figure 3C−3D). In addition, miR-192-5p showed differences in median concentration in between early and late stage HCCs (Figure 3C). These findings indicate that miR-192-5p and miR-29a-3p have good value for early event detection, specifically for miR-192-5p in predicting tumor recurrence and metastasis.

**Figure 4:**
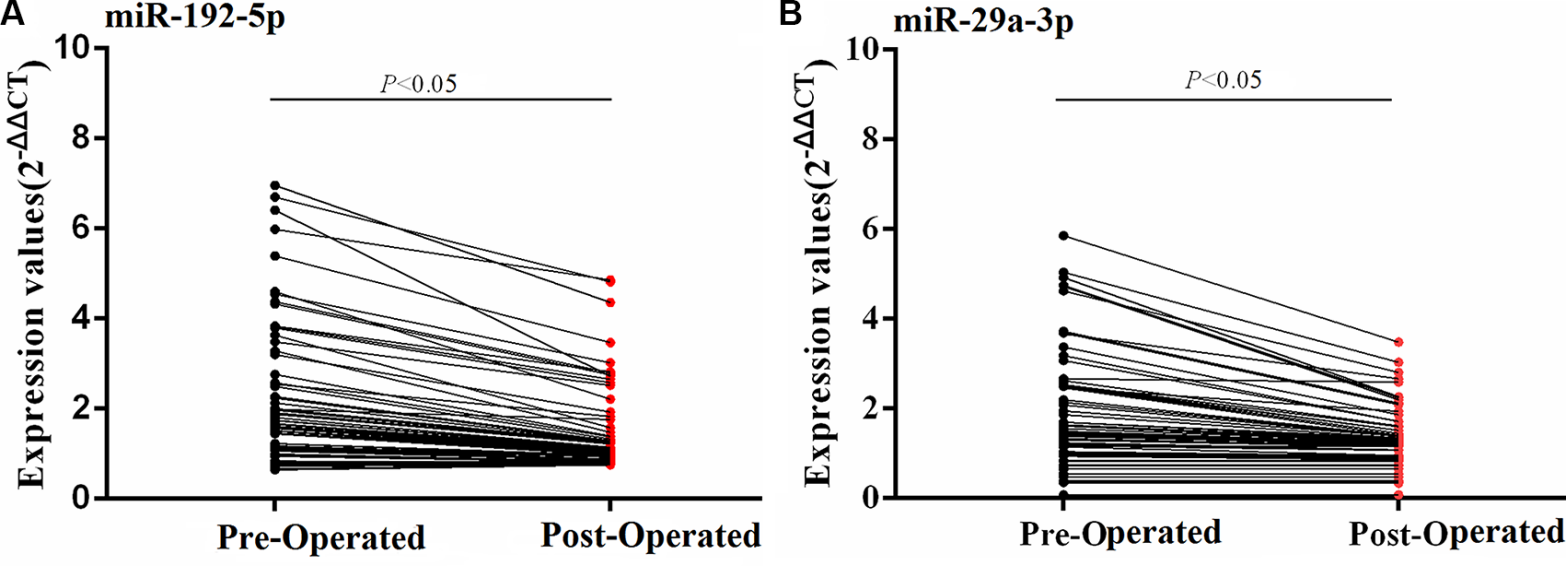
Dynamic expression changes of serum miRNAs between pre- and post-operated HCCs in the training set The serum miRNAs concentration of pre- and post-operated HCCs were compared using Wilcoxon matched-pairs signed rank test.

In HCC patients, a decrease of the 2 serum miRNAs concentration between pre- and post-operated HCCs was observed in HCCs serum in the training set (Figure 4A–4B).

### Risk model for predicting survival in HCCs from the training set

Finally, 3 variables, including BCLC stage, miR-192-5p and miR-29a-3p, were analyzed in a multivariate risk model to calculate the PI_OS_ score, while another model combining BCLC stage, miR-192-5p and AFP was analyzed to calculate the PI_PFS_ score. The scores were calculated by the following equations (method described in Statistical analysis and Supplementary Materials): PI_OS_ = 2.19 (0 or 1) + 1.45 (0 or 1) + 1.81(0 or 1); PI_PFS_ = 1.89 (0 or 1) + 1.07 (0 or 1)+ 0.69 (0 or 1). The PI_OS_ and the PI_PFS_ score had a range of 0−5.45 and 0−3.65, respectively. Consequently, patients with low-, medium- and high-risk had OS/PFS-predictor scores of < 1.81/0.69, 1.81−3.64/0.69−1.89 and > 3.64/1.89.

**Figure 5:**
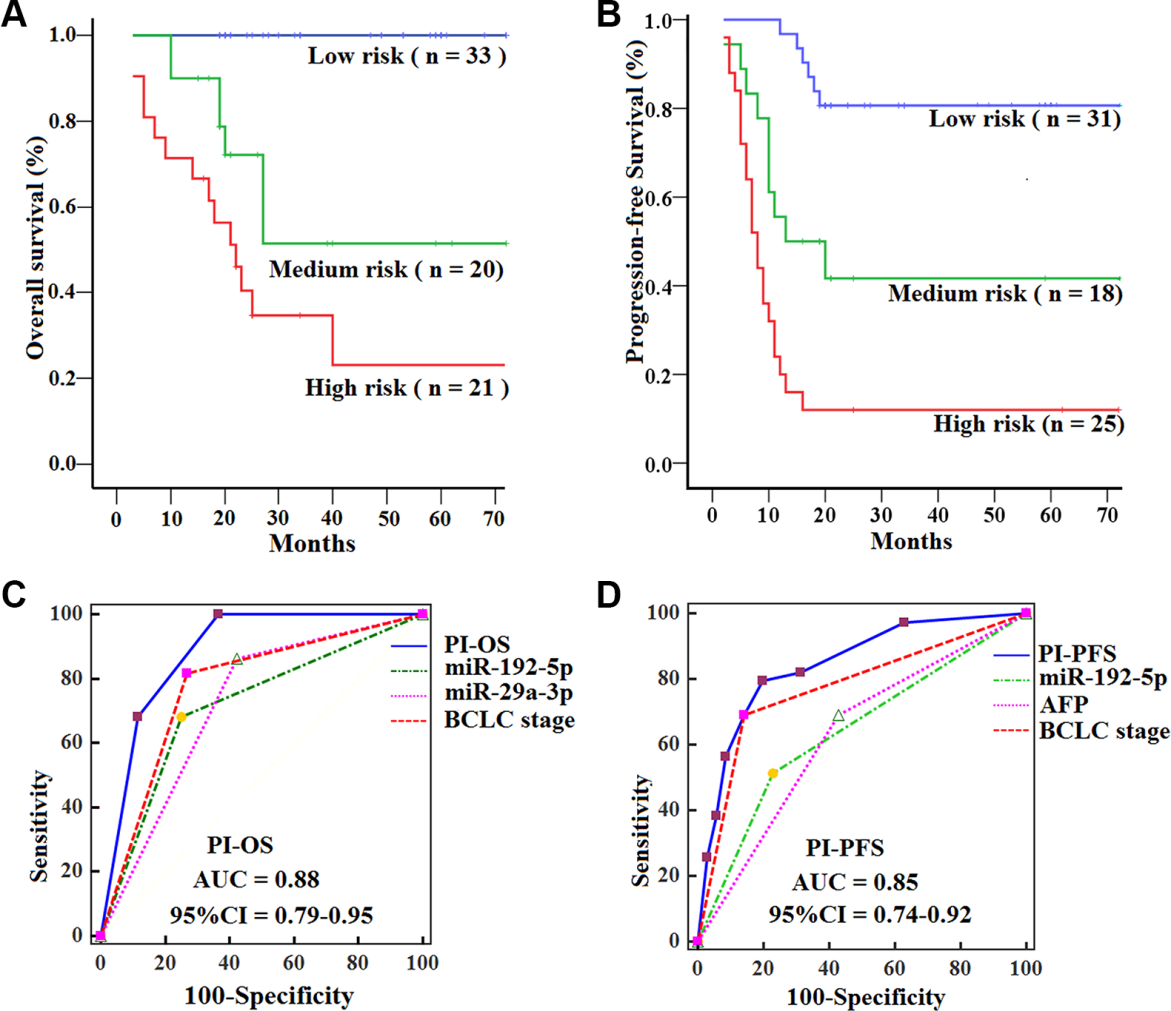
Kaplan-Meier curves of OS and PFS for HCCs with subtypes in the training set, with comparisons of the sensitivity and specificity for HCCs prediction of survival by PI_OS_, PI_PFS_, clinical variables and miRNAs OS (**A**) and PFS (**B**) were calculated in 74 HCCs subdivided by PI into low-, medium- and high-risk subtypes. Log-rank test was used to calculate *P* value. AUCs of PI_OS_, PI_PFS_, clinical variables and miRNAs for OS (**C**) and PFS (**D**) were analyzed with ROC curves.

HCCs in the low-risk group had significantly longer OS (log-rank test, *P* = 0.000; *P* = 0.000) and PFS (*P* = 0.004; *P* = 0.000) than those in the medium- and high-risk group, and HCCs in the medium-risk group had longer OS (*P* = 0.038) and PFS (*P* = 0.009) than those in the high-risk group (Figure 5A−5B). In addition, ROC analyses revealed that the PI showed better survival prediction than the other prognostic characteristics as PI_OS_ and PI_PFS_ had the best AUCs. (all *P* < 0.05) (Figure 5C−5D). Multivariate analyses also demonstrated that PI (PI_OS_ and PI_PFS_) was the only significantly independent prognostic predictor for OS (HR = 5.2, 95% CI 2.6–10.9; *P* = 0.000) and PFS (HR = 3.1, 95% CI 2.0–4.7; *P* = 0.000) of HCCs in the training set.

### Validating the risk model of PI (PI_OS_, PI_PFS_) in the validation set

**Figure 6:**
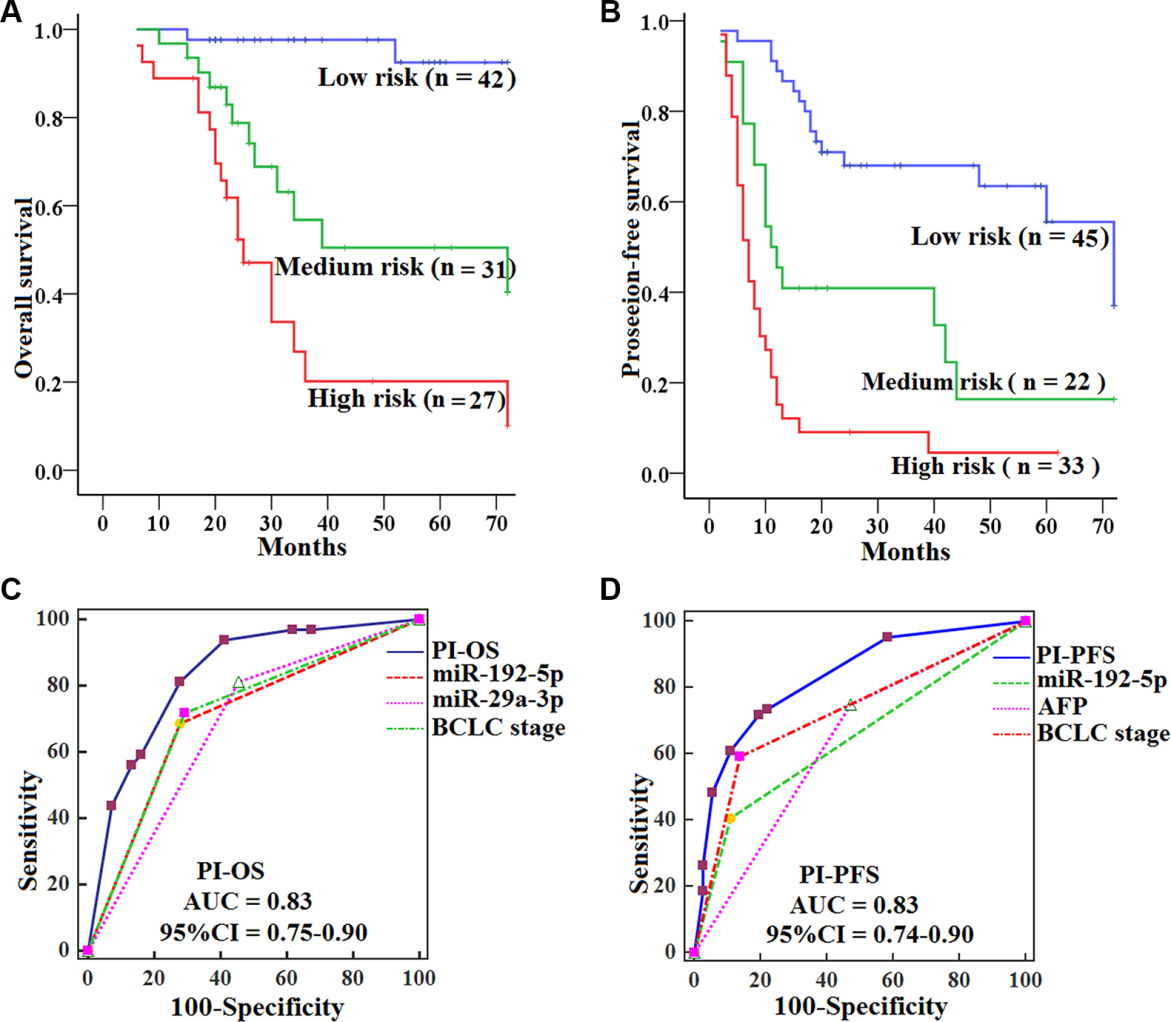
Kaplan-Meier curves of OS and PFS for HCCs by subtypes in the validation set, with comparisons of the sensitivity and specificity for HCCs prediction of survival by PI_OS_, PI_PFS_, clinical variables and miRNAs OS (**A**) and PFS (**B**) were calculated in 100 HCCs subdivided by PI into low-, medium- and high-risk subtypes. Log-rank test was used to calculate *P* value. AUCs of PI_OS_, PI_PFS_, clinical variables and miRNAs for OS (**C**) and PFS (**D**) were analyzed with ROC curves.

To validate the prognostic value of the PI score, we quantified the levels of serum miR-192-5p and miR-29a-3p in a validation set of 100 HCCs and 70 HCs samples. We then calculated the risk score for each patient and classified patients into high-, medium- and low-risk groups using the same formulas and method as used in the training set. Similar to what was seen in the training set, HCCs in the low-risk group had significantly longer OS (log-rank test, *P* = 0.000; *P* = 0.000) and PFS (*P* = 0.001; *P* = 0.000) than those in the medium- and high-risk group, and HCCs in the medium-risk group had longer OS (*P* = 0.016) and PFS (*P* = 0.004) than those in the high-risk group (Figure 6A−6B). Likewise, the PI_OS_ and PI_PFS_ had the best AUCs among prognostic factors in the validation set, showed a better survival prediction than the other prognostic characteristics by ROC analyses (all *P* < 0.05) (Figure 6C−6D), and were independent prognostic predictors for OS (HR = 3.8, 95% CI 2.3–6.4; *P* = 0.000) and PFS (HR = 2.7, 95% CI 2.0–3.7; *P* = 0.000) of HCCs by multivariate analyses.

### The 2 miRNAs are overexpressed in HCC tissues

**Figure 7:**
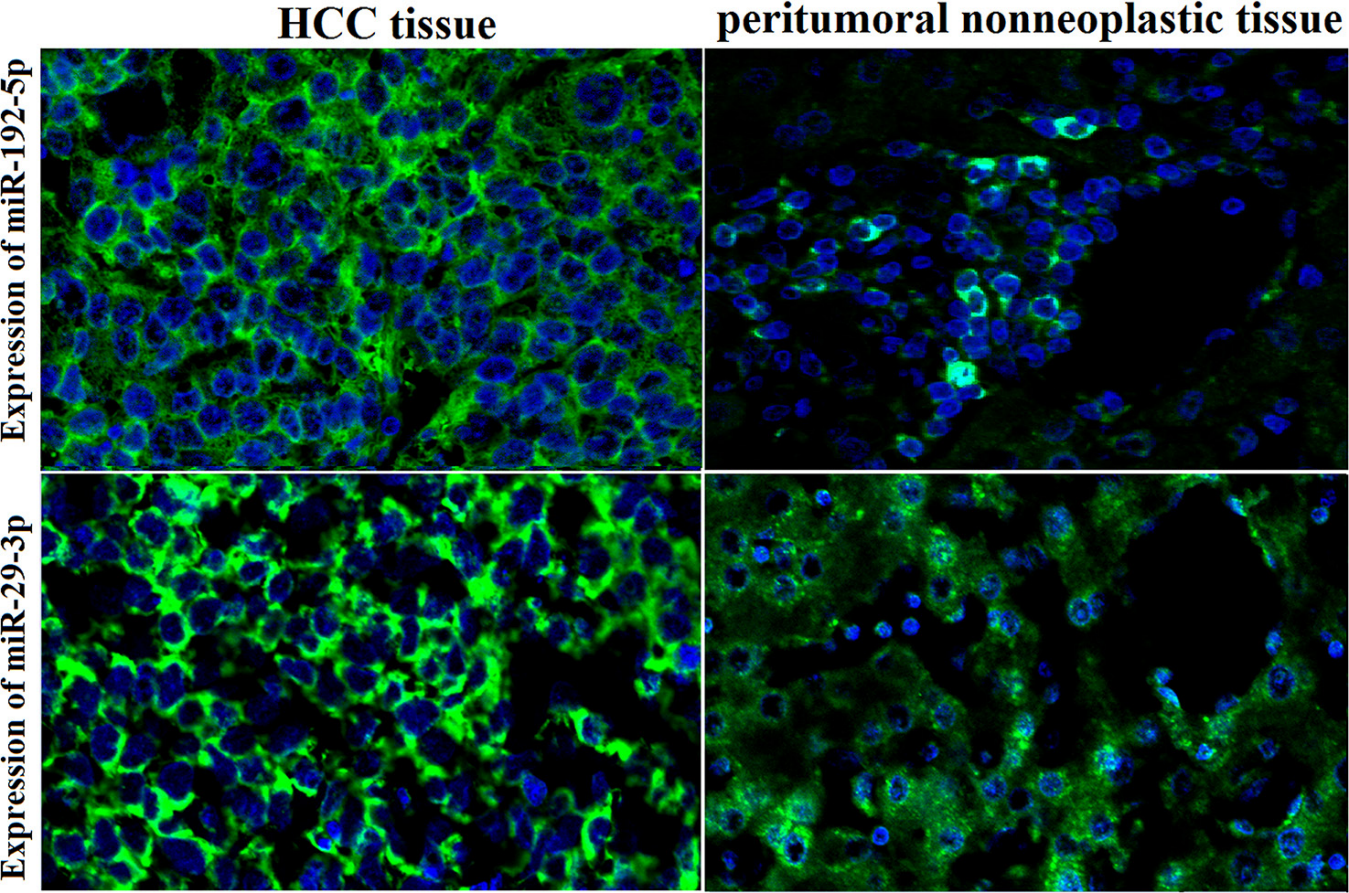
High expression of miR-192-5p and miR-29a-3p in HCC tissues by FISH An increase in miR-192-5p and miR-29a-3p staining intensity was observed in HCC tissues compared with corresponding normal adjacent tissues by FISH. Positive *in situ* hybridization signals are visualized in green, while blue depicts DAPI nuclear stain (×400 magnification).

In HCC patients, an increase in miR-192-5p and miR-29a-3p staining intensity was observed in HCC tissues compared with corresponding normal adjacent tissues by FISH (Figure 7).

## DISCUSSION

We identified miR-192-5p and miR-29a-3p as inversely associated with survival and progression in HCCs. We found that a combination of miRNA expression levels and clinical variables, had a better prognostic value than BCLC stage alone.

In the clinic, HCCs with the same stage who receive similar treatment regimens often show large variations in disease outcome, indicating that the current staging system may have reached its limitations for prognostic assessment. Staging in HCC patients is challenging because it requires staging and grading of both tumor and the chronic liver disease. Therefore, novel markers that reflect the aggressiveness of the disease would complement the current set of prognostic and treatment algorithms.

Circulating miRNAs are stably detectable in serum or plasma [11, 23, 24] and have potential as novel prognostic biomarkers in a variety of cancers [15–17, 25–27]. We identified a risk model that was significantly associated with patient survival. Compared to patients with low- and medium-risk scores, HCCs with high-risk scores had reduced OS and PFS, verifying the prognostic value of serum miRNAs and the risk model of PI score.

Several publications suggest significant roles for miRNAs in the evaluation of cancer prognosis and diagnosis, such as lung cancer and HCC [28–30]. In the present study, serum miR-192-5p and miR-29a-3p as new prognostic parameters in HCCs are independent of classical BCLC stage. Therefore, we combined serum miRNAs and clinical variables to develop a risk model [15, 25–27, 30–34] of PI score, and the combination of serum miRNAs and BCLC stage had a better prognostic value than BCLC stage alone. So, the serum miRNA signature can add prognostic value to the BCLC stage system. As a result, HCCs could be stratified and distinguished into different risk groups, and could be treated with different approaches or intensities to improve their disease outcomes.

Logistic regression with penalized estimates may be used to develop prognostic models for binary outcomes, especially when limited data are available. Discrimination, calibration, and overall performance were taken into account in the construction of models [35]. ROC curve analysis was used to quantify a concordance statistic (c) in logistic regression [36]. The risk model was applied successfully to predict patient survival in lung cancer [30] and diffuse large B-cell lymphoma [31]. In our study, the PI divided HCCs into 3 groups correlated to differential survival. Such information not only may improve the predictive performance of the model, but also strengthen its clinical credibility.

Deep sequencing can be focused on both discovering novel miRNAs and acquiring a highly quantitative estimate of known individual miRNA species [37]. Developing minimally invasive methods including deep sequencing in the field of miRNAs for HCC diagnosis and prognosis is of great interest. Thus, a serum miRNA signature in HCCs, as shown in the present study, might be of great clinical interest as a routine testing procedure.

Decrease of both miR-192-5p and miR-29a-3p has been shown in HCC cells in several studies [8, 38–40], but two other studies have identified these same miRNAs as HCC diagnostic markers increased in the circulation of HCC patients [19, 41]. Our studies are in agreement with the literature on diagnostic circulating miRNA panels which indicate that miR-192-5p and miR-29a-3p have considerable clinical credibility for HCC diagnosis. In particular, miR-192-5p is expressed differentially in early and late stage HCC, and is a promising biomarker for HCC surveillance.

The American Association for the Study of Liver Diseases Practice Guidelines (July 2010) discarded AFP for surveillance and diagnosis. Therefore, there is a need for novel markers that would serve as a reliable method for early detection and postsurgical progression. This need might be well fulfilled with serum miR-192-5p and serum miR-29a-3p as alternative biomarkers which may be particularly helpful for AFP-negative HCCs

However, there are some limitations in our study. First, in the discovery stage, if we had compared the expression of serum miRNAs of the controls with that of the prognosis well and the poor HCC patients, it would be better in experimental design. Second, currently no established internal reference exists for circulating miRNAs. Non-human spike-in miRNAs, such as from *Caenorhabditis elegans* [42, 43] have been suggested as alternative references. Third, the two-miRNA signature was identified from Chinese patients at a single medical center. Fourth, the origins and mechanisms leading to the generation of serum miRNAs remain unclear. Finally, further studies are warranted to evaluate these miRNAs, to characterize the molecular mechanisms underlying miRNA function, and to discover potential therapy targets for HCC.

In conclusion, distinct serum miRNA profiles associated with survival exist in HCCs. Additionally, these serum miRNAs may be a robust predictor of prognosis for HCC, which may lead to more personalized therapy. Moreover, deep sequencing followed by qPCR validation may be a successful strategy to determine biomarkers in circulation.

## MATERIALS AND METHODS

### Study participants

All samples were obtained from confirmed cases of HCC at the time of diagnosis from the Sun Yat-sen University Cancer Center (SYSUCC) (Guangzhou, China). For the discovery stage of this study, we screened serum miRNAs in a set of 50 HCCs and 50 HCs consecutively obtained between January 2009 and April 2009. For subsequent study, (Training stage and Validation stage), we used randomly selected samples obtained between May 2009 and December 2013 from 174 HCCs, 43cirrhosis patients and 130 HCs to test and validate miRNA expression at SYSUCC. HCs were individuals who came in for a routine physical examination and were found to be cancer-free. The characteristics of the 174 HCC patients are summarized in Table 3. The inclusion criteria for the study were a first time diagnosis of HCC with no history of other tumors and a follow-up time of > 1 year at the completion of this study. Informed consent was obtained for all participants. This study was approved by the medical research ethics committee of SYSUCC. All patients were treated with hepatic resection, local ablation or a combination of both.

**Table 3:** Clinicopathological features and follow-up data for 174 patients in the training and validation data sets

Variable	Training (*n* = 74)	Validation (*n* = 100)
	No.	No.
Age,years		
Median	52	51
Mean	51	51.5
Follow-up,months		
Median	27	28
Mean	35	36
Sex		
Male	63	84
Female	11	16
AFP^1^		
≤ 200 ng/ml	44	58
> 200 ng/ml	30	42
HBV-DNA		
≤ 1000 IU/ml	26	37
> 1000 IU/ml	48	63
Child-Pugh^2^		
A	66	88
B	8	12
Vascular invasion (+/−)		
+	8	12
−	66	88
Tumor size		
≤ 3 cm	28	35
> 3 cm	46	65
BCLC stage^3^		
0	15	17
A	27	40
B	24	31
C	8	12
Treatment regimen		
Resection	24	25
Local ablation^4^	25	30
Resection + Local ablation	25	45

AFP, alpha fetoprotein; Child-Pugh, a model to evaluate liver function;

BCLC, Barcelona Clinic Liver Cancer stage; Including transarterial chemoembolisation (TACE) and radiofrequency ablation (RFA).

### Serum preparation and RNA extraction

Venous blood was incubated at room temperature for 1 hr, centrifuged at 500 g for 10 min and then 10,000 g for 30 min at 4°C to separate the serum. The supernatant was transferred to fresh RNase-free tubes and stored at −80°C until use. We extracted total RNA using TRIzol^®^ LS reagent (Life Technologies, Carlsbad, CA) following the manufacturer's recommendations. More detailed information is provided in the Supplementary Materials.

### Small RNA library construction and sequencing

We created 2 small RNA libraries according to the protocol published by Morin et al. [20]. To fully investigate the differences in serum miRNA profiles between HCCs and HCs in the discovery stage, sequencing was performed on the Illumina/HiSeq 2000 platform [Beijing Genomics Institute (BGI)]. More details are provided in the Supplementary Materials.

### Bioinformatics analysis of high-throughput data

Detailed information is provided in the Supplementary Materials.

### Analysis of differential miRNA expression

To identify miRNAs differentially expressed between HCCs and HCs in the discovery stage, we used the Bayesian method developed by Audic and Claverie [21]. miRNAs displaying at least a 2-fold change difference between the 2 groups were selected for further investigation. A detailed description of this procedure is shown in the Supplementary Materials.

### Mature miRNA qPCR

To find a miRNA signature with which to construct a risk model in the training set, SYBR^®^Green based quantitative PCR (qPCR) reactions were performed on the 7500 Real-time PCR systems (Applied Biosystems, Carlsbad, CA) to validate 12 mature up-regulated miRNAs screened by sequencing (see Supplementary Table S3). The miRNA signature was further confirmed in the validation set by qPCR method. The detailed method is described in the Supplementary Materials.

### Locked nucleic acid (LNA)-based *in situ* hybridization for miRNA in HCC

To study the spatial and temporal expression of miRNAs with high sensitivity and resolution, the miRNA fluorescein *in situ* hybridization (FISH) protocol were optimized and performed. More details are provided in the Supplementary Materials.

### Statistical analysis

To compare the sequencing data of the HCCs and HCs, we used the Wilcoxon–Mann–Whitney test. For the data obtained by qPCR, either the Mann-Whitney unpaired test or the Kruskal–Wallis test was used for the comparison between HCC and control (cirrhosis patients and healthy controls). We compared the serum miRNAs concentration of pre- and post-operated HCCs using Wilcoxon matched-pairs signed rank test. The levels of all miRNAs with significance values < 0.05 were considered statistically significant.

We defined overall survival (OS) and progression free survival (PFS) as the period between the date of diagnosis and recurrence/metastasis, death, or last day of follow-up. Survival curves were estimated by use of the Kaplan–Meier method with the log-rank test. We used a multivariate Cox regression analysis to investigate whether the selected miRNAs were independent prognostic factors of OS and PFS in HCCs.

For each miRNA expression profile, we plotted the sensitivity and specificity of each outcome as a predictor of death for HCC with receiver operating characteristic (ROC) curve analysis. We used ROC curves as an accuracy index for assessing the predictive power as well as evaluating the diagnostic performance of each variable. We used the ROC curves to select cutoff scores for dichotomizing each predictor according to the maximum area under the ROC curve (AUC). The cutoff scores were generated with MedCalc software, version 12.2.1.

Penalized maximum likelihood estimation was applied to select the optimal variables to establish a multivariate risk model for evaluating HCC patient outcome. To evaluate the joint efficacy of this multivariate risk model, we introduced a prognostic index (PI). In general, we defined the PI score in terms of the logistic regression model: PI = logit{*Y* = 1 | *X*} = *β*_0_ + Σ*β_i_x_i_* [22], where *Y* is a binary outcome variable (0 or 1), *β*_0_ is an intercept, and denotes the regression coefficients associated with the *β_i_* design matrix *X* of covariables *i*. Specifically, in our study we calculated the PI score according to the formula: PI = *β*_1_*x*_1_ + *β*_2_*x*_2_ + *β*_3_*x*_3_, where *β* is a variable assignment indicated in univariate Cox regression analyses for HCCs in the training set (see Supplementary Table S2). A ROC curve was generated for further analysis of the prognostic value of PI. We used the maximum Youden index to obtain optimal cutoff values for PI_OS_ and PI_PFS_ for prognostic assessment in HCCs. Subsequently, ROC curve analyses for each subgroup were applied to calculate the sensitivity and specificity of PI.

Two-sided *P* values of < 0.05 were considered to indicate statistical significance. Statistical analyses were carried out by use of SPSS 17.0.

## Supplementary Materials Figures and Tables


