# Supplementary material for: Serum microRNA profiles as prognostic biomarkers for HBV-positive hepatocellular carcinoma
Source: Oncotarget. 2016 Jun 15;7(29):45637–48. doi: 10.18632/oncotarget.10082 (PMC5216748; doi:10.18632/oncotarget.10082)
Supplement: Supplementary file 1 [file oncotarget-07-45637-s001.pdf]

Serum microRNA profiles as prognostic biomarkers for HBV-positive hepatocellular carcinoma

Supplementary Materials

SUPPLEMENTARY METHODS

RNA extraction, small RNA library construction, and sequencing

Total RNA was isolated from 250 µl of serum with 750 µl of TRIzol®LS reagent (Life Technologies, Carlsbad, CA). Synthetic C. elegans miRNA, cel-miR-39 (Life Technologies, Carlsbad, CA), was used to normalize possible sample-to-sample variation in RNA isolation as described previously [1]. The extracted total RNA was allowed to air dry at 25°C and re-suspended in 25 µl of diethylpyrocarbonate (DEPC) water (Ambion, Austin, TX, USA) after the final precipitation step. RNA concentration and purity were measured at 260 and 280 nm using a spectrophotometer (NanoDrop Technologies, Wilmington, DE, USA).

In terms of small RNA library preparation, small RNAs (18–30 nt) were obtained from total RNA, and 5′ and 3′ adaptors were ligated to the small RNAs. The adaptor-ligated RNAs were subsequently transcribed into cDNA by RT-PCR using the adaptor primers for 17 cycles and the fragments around 90 base-pairs (small RNA + adaptors) were isolated from agarose gels, and the samples were amplified by PCR. The PCR products were purified and directly subjected to cluster generation and sequencing analysis using the Illumina/HiSeq 2000 at the Beijing Genomics Institute (BGI, Shenzhen, China). The image files generated by the sequencer proceeded to yield digital-quality data.

Bioinformatics analysis of high-throughput data

After removal of adaptors, insert, poly (A), and shorter RNAs than 18nt, clean and high quality reads were obtained according to previous literatures [2, 3]. These data indicated that the high quality of reads were appropriate for sequencing analysis. These reads were mapped to the reference genome by forcing perfect alignments beginning at the first nucleotide and retaining the longest region of each read that could be aligned to the reference genome, along with all alignment positions.

To identify conserved miRNAs, the filtered sequences contaminated by rRNA, tRNA, snRNA, and snoRNA were initially used to search miRBase release v17.0 (http://www.mirbase.org/) and utilizing BLASTN for the comparison of the candidate miRNAs to the reference miRNA sequences. The remaining putative novel miRNAs were mapped with BLAST against known miRNA from miRBase v17, ncRNA from Ensembl. The criterion was implemented according to a reported miRNA protocol [4]. We excluded sequences that aligned with more than 90% of their length (allowing 1 mismacth) to any of the ncRNA sequences.

Analysis of differentially expressed miRNAs

The Bayesian method was developed for analysis of digital gene expression profiles and accounts for the sampling variability of reads with low counts [5]. Let us denote $P(x)$ the probability to observe x sequence tags of the same gene (i.e., from the 3′ end of the same transcript) when N cDNA clones are picked randomly. For each transcript representing a small (i.e., less than 5%) fraction of the library and $N \geq 1000$, $P(x)$ will closely follow the Poisson distribution (formula 1–1). The analysis process was shown in detail: (1) Normalize the expression of miRNA between the two groups to obtain the expression of transcript per million. Normalized expression (NE) = Actual miRNA count/total count of clean reads. (2) Calculate fold change and P value from the normalized expression. Then the log2 ratio and scatter plot were generated. Fold change formula: Fold change = log2 (HCC_NE/HC_NE). P value formula was indicated the formula (1–2). The x and y represented normalized expression levels, and the $N1$ and $N2$ represented total count of clean reads of a given miRNA in two groups, respectively.

$$p(x) = \frac{e^{-\lambda}\lambda^{x}}{x!} \qquad \text{1-1}$$

$$p(y \mid x) = \left(\frac{N_2}{N_1}\right)^{y} \frac{(x+y)!}{x!y!\left(1+\frac{N_2}{N_1}\right)^{(x+y+1)}}$$

$$C(y \leq y_{\min} \mid x) = \sum_{y=0}^{y \leq y_{\min}} p(y \mid x)$$

$$D(y \leq y_{\max} \mid x) = \sum_{y=y_{\max}}^{\infty} p(y \mid x) \qquad \text{1-2}$$

Mature miRNA quantitative real time PCR (qPCR)

SYBR®Green based quantitative PCR (qPCR) analysis was carried out on the 7500 Real-time PCR systems (Applied Biosystems, Carlsbad, CA). A stem-loop primer based qPCR assay was used to verify 12

mature up-regulated miRNAs screened by sequencing. Each sample was analyzed in triplicate and *C. elegans* spiked-in miRNAs *cel-miR-39* was used for serum RNA normalization control. The relative expression of each miRNA was determined using the equation $2^{-\Delta CT}$, where ΔC_T = mean $C_{T\,\text{miRNA}}$ – mean $C_{T\,\text{control}}$ (where C_T values were the threshold cycle for each sample). The fold change values were calculated relative to the average expression in serum of HCs by using the equation $2^{-\Delta\Delta CT}$, in which $\Delta\Delta C_T$ = (mean $C_{T\,\text{cancer}}$ – mean $C_{T\,\text{control}}$) − (mean $C_{T\,\text{health}}$ – mean $C_{T\,\text{control}}$) [6].

LNA-based FISH for miRNA

This assay was performed according to the manufacturer's protocol (Exiqon, Vedbaek,Denmark). Briefly, thin sections (4 um thick) of paraffin-embedded specimens were deparaffinized with xylene and rehydrated with graded ethanol dilution. Sections were treated with 0.05% trypsin at room temperature for 15 minutes and re-fixed in 4% paraformaldehyde for 10 minutes. The slides were prehybridized in a hybridization solution at 51°C for 2 hours. Subsequently, 20 nmol/L of a locked nucleic acid-modified, 5′digoxigenin (DIG)-labeled oligonucleotide probe complementary to miRNAs or a scrambled control probe was added to 100 ml of the hybridization solution and hybridized at a temperature of 51°C overnight. The sections were rinsed twice in 2× standard saline citrate, followed by three washes of 20 minutes at 50°C in 50% formamide/2× standard saline citrate. Then, the samples were washed five times in PBS/0.1% Tween-20 and blocked in blocking solution (2% sheep serum, 2 mg/ml bovine serum albumin in phosphate buffered saline with Tween-20) at room temperature for 1 hour. An anti-DIG antibody (1:1000; Abcam, Cambridge, MA, USA) was applied, and the sections were incubated at 4°C overnight. *In situ* hybridization signals were detected using the tyramide signal amplification system (PerkinElmer, USA) according to the manufacturer's instructions. Slides were mounted in Prolong Gold containing 4′,6-diamidino-2-phenylindole (DAPI) (Invitrogen) and analyzed with an Confocal laser scanning microscopy equipped with a Jenoptik camera and VideoTesT-FISH 2.0 software (Olympus).

REFERENCES

1. Mitchell PS, Parkin RK, Kroh EM, Fritz BR, Wyman SK, Pogosova-Agadjanyan EL, Peterson A, Noteboom J, O'Briant KC, Allen A, Lin DW, Urban N, Drescher CW, et al. Circulating microRNAs as stable blood-based markers for cancer detection. Proceedings of the National Academy of Sciences of the United States of America. 2008; 105: 10513–10518.
2. Morrissy AS, Morin RD, Delaney A, Zeng T, McDonald H, Jones S, Zhao Y, Hirst M, Marra MA. Next-generation tag sequencing for cancer gene expression profiling. Genome research. 2009; 19:1825–1835.
3. t Hoen PAC, Ariyurek Y, Thygesen HH, Vreugdenhil E, Vossen RHAM, de Menezes RX, Boer JM, van Ommen GJB, den Dunnen JT. Deep sequencing-based expression analysis shows major advances in robustness, resolution and inter-lab portability over five microarray platforms. Nucleic Acids Research. 2008; 36.
4. Sdassi N, Silveri L, Laubier J, Tilly G, Costa J, Layani S, Vilotte JL, Le Provost F. Identification and characterization of new miRNAs cloned from normal mouse mammary gland. BMC genomics. 2009; 10:149.
5. Audic S, Claverie JM. The significance of digital gene expression profiles. Genome Res. 1997; 7:986–995.
6. Gramantieri L, Ferracin M, Fornari F, Veronese A, Sabbioni S, Liu CG, Calin GA, Giovannini C, Ferrazzi E, Grazi GL, Croce CM, Bolondi L, Negrini M. Cyclin G1 is a target of miR-122a, a microRNA frequently down-regulated in human hepatocellular carcinoma. Cancer research. 2007; 67:6092–6099.

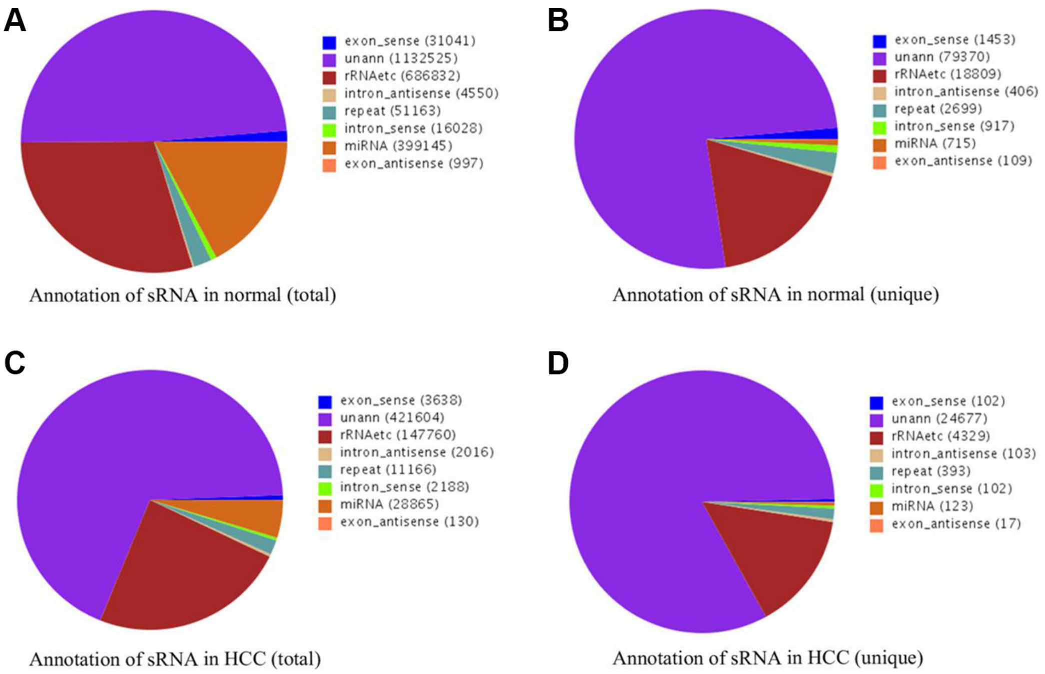

Supplementary Figure S1: All annotated small RNAs with mapping statistics in HCC patients and HCs serum samples from the discovery set. Total, small RNA weighted by total read counts in healthy controls and HCC patients (**A** and **C**); unique, small RNA weighting in healthy controls and HCC patients after removal of low quality reads and redundancy (**B** and **D**). Abbreviations: HCC, hepatocellular carcinoma; HCs, healthy controls.

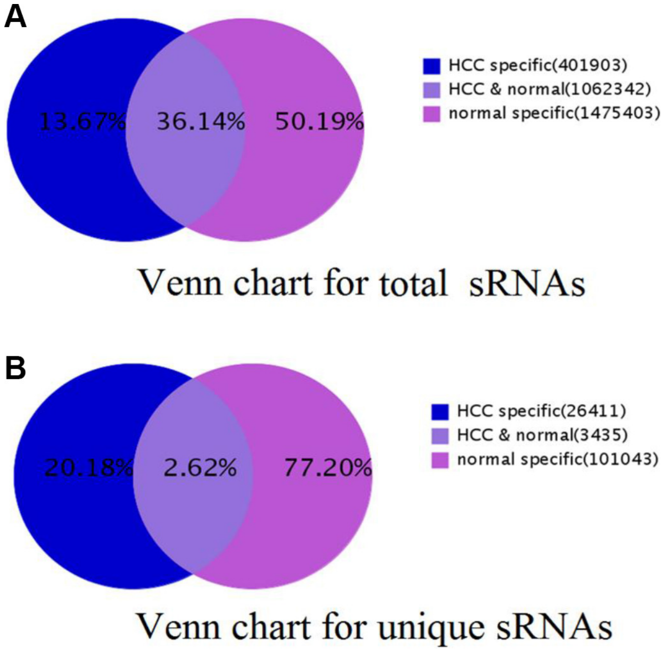

Supplementary Figure S2: Venn diagram showing the overlap in each small RNAs library. The mediate color indicated the overlaps small RNA reads between HCC patients and HCs. (**A**) description of the overlaps when yielding small RNAs by total reads; (**B**) description of the overlaps when yielding unique sRNAs after removal of low quality reads and redundancy. Abbreviations: HCC, hepatocellular carcinoma; HCs, healthy controls.

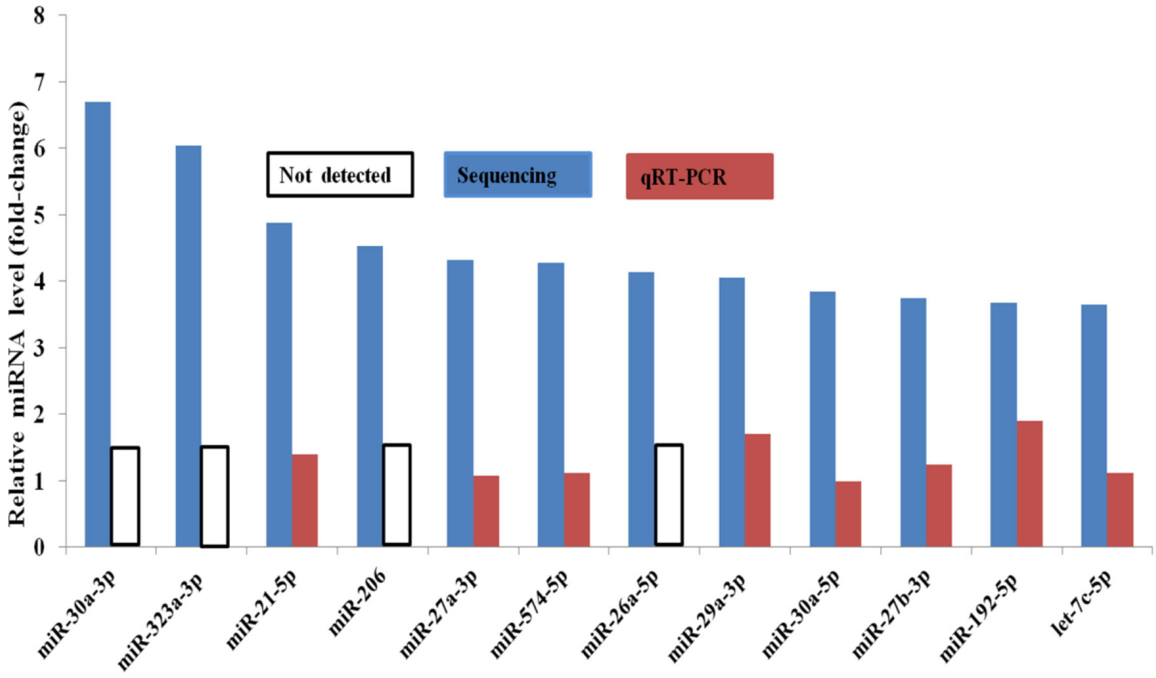

Supplemental Figure S3: Comparison of differentially expressed miRNAs determined by deep sequencing and qPCR. The sequencing relative quantity (RQ) was calculated by the miRNA signal intensities in HCC patients (*n* = 50) *versus*. HCs (*n* = 50) from the discovery cohort. The qPCR RQ is calculated as RQ = $2^{-\Delta\Delta CT}$ (see Supplemental methods), where is RQ > 1 indicating up-regulation in HCC, RQ < 1 indicating down-regulation in HCC comparing to HC. Abbreviations: HCC, hepatocellular carcinoma; HCs, healthy controls.

Supplementary Table S1: 146 known human serum miRNAs screened by Illumina/HiSeq 2000 platform from the discovery set of 50 HCs[1] and 50 HCC[2] patients. See Supplementary_Table_S1

Supplemental Table S2: Univariate Cox regression analysis of OS[1] and PFS[2] according to prognostic factors of 74 HCC[3] patients in the training set

	OS				PFS			
**Variables**	**HR[4]**	**95% CI[5]**	***P***	**β[6]**	**HR**	**95% CI**	***P***	**β**
Gender								
Male	1	reference			1	reference		
Female	0.4	(0.1–1.4)	0.154	0	0.9	(0.3–2.9)	0.87	–0.098
Age (years)								
≤ 49	1	reference			1	reference		
> 49	0.6	(0.2–1.3)	0.186	–0.567	0.7	(0.4–1.3)	0.244	–0.387
AFP[7]								
≤ 36 ng/ml	1	reference			1	reference		
> 36 ng/ml	1.2	(0.5–2.7)	0.718	0.155	2.0	(1.0–4.0)	0.046*	0.693
HBV-DNA								
≤ 1000 IU/ml	1	reference			1	reference		
> 1000 IU/ml	0.7	(0.3–1.7)	0.500	–0.292	0.2	(0.3–1.2)	0.636	–0.452
Histological grade								
G1–G2	1	reference			1	reference		
G3	0.7	(0.2–2.5)	0.641	–0.290	0.6	(0.2–1.6)	0.615	–0.486
Tumor size								
≤ 5 cm	1	reference			1	reference		
> 5 cm	6.1	(2.4–15.1)	0.000*	1.808	4.6	(2.5–8.9)	0.000*	1.536
Vascular invasion								
–	1	reference			1	reference		
+	7.4	(2.8–19.6)	0.000*	2.014	4.5	(2.0–10.0)	0.000*	1.509
BCLC[8] stage								
0−A	1	reference			1	reference		
B−C	8.9	(3.5–31.2)	0.000*	2.191	6.6	(3.3–13.3)	0.000*	1.886
miR-192-5p								
≤ 2.24	1	reference			1	reference		
> 2.24	4.3	(1.7–10.5)	0.002*	1.449	2.9	(1.5–5.5)	0.001*	1.066
miR-29a-3p								
≤ 1.37	1	reference			1	reference		
> 1.37	6.1	(1.8–20.6)	0.004*	1.805	2.1	(1.1–4.2)	0.028*	0.753

Notes: 1. OS, overall survival; 2. PFS, progression free survival; 3. HCC, hepatocellular carcinoma; 4. HR, hazard ratio; 5. CI, confidence interval; 6. β, regression coefficient; 7. AFP, alpha fetoprotein; 8. BCLC, Barcelona Clinic Liver Cancer stage, Early stage: (0−A), Late stage: (B −C); $*P < 0.05$.

Supplementary Table S3: 12 up-regulated serum microRNAs qPCR assay

microRNA	Sequence
miR-30a-3p	CUUUCAGUCGGAUGUUUGCAGC
miR-323a-3p	CACAUUACACGGUCGACCUCU
miR-21-5p	UAGCUUAUCAGACUGAUGUUGA
miR-206	UGGAAUGUAAGGAAGUGUGUGG
miR-27a-3p	UUCACAGUGGCUAAGUUCCGC
miR-574-5p	UGAGUGUGUGUGUGUGAGUGUGU
miR-26a-5p	UUCAAGUAAUCCAGGAUAGGCU
miR-29a-3p	UAGCACCAUCUGAAAUCGGUUA
miR-30a-5p	UGUAAACAUCCUCGACUGGAAG
miR-27b-3p	UUCACAGUGGCUAAGUUCUGC
miR-192-5p	CUGACCUAUGAAUUGACAGCC
let-7c-5p	UGAGGUAGUAGGUUGUAUGGUU
cel-miR-39[1]	UCACCGGGUGUAAAUCAGCUUG
RNU6B[2]	CGCAAGGAUGACACGCAAAUU;<br>CGUGAAGCGUUCCAUAUUUUU

Notes: 1. as internal reference for circulating miRNAs; 2. as internal reference for tissue miRNAs.
